# Antibiotic-Resistant *Escherichia coli* and *Salmonella* from the Feces of Food Animals in the East Province of Rwanda

**DOI:** 10.3390/ani11041013

**Published:** 2021-04-03

**Authors:** Rosine Manishimwe, Paola M. Moncada, Vestine Musanayire, Anselme Shyaka, H. Morgan Scott, Guy H. Loneragan

**Affiliations:** 1 Department of Animal and Food Sciences, Texas Tech University, Lubbock, TX 79415, USA; paola-melisa.moncada@ttu.edu; 2 Department of Veterinary Medicine, University of Rwanda, Nyagatare 56, Rwanda; shyakaa@gmail.com; 3 Rwanda Veterinary Service Department, Rwanda Agriculture and Animal Resources Development Board, Ministry of Agriculture, Kigali 5016, Rwanda; Vestine.musanayire@rab.gov.rw; 4 Department of Veterinary Pathobiology, Texas A&M University, College Station, TX 77843, USA; hmscott@cvm.tamu.edu; 5 School of Veterinary Medicine, Texas Tech University, Amarillo, TX 79106, USA; guy.loneragan@ttu.edu

**Keywords:** antibiotic resistance, food animals, *E. coli*, *Salmonella*, Rwanda

## Abstract

**Simple Summary:**

A paucity of information on antimicrobial resistance in animals in Rwanda prompted us to conduct this study, the objective of which was to estimate the prevalence of antibiotic resistance among *Escherichia coli* and *Salmonella* from the feces of cattle, goats, pigs, and poultry. We found that resistance to tetracycline, ampicillin, and streptomycin were the most frequent among non-type-specific *E. coli* isolates. Resistance to chloramphenicol, quinolone-based antibiotics, amoxicillin–clavulanic acid, and azithromycin were also observed among these bacterial isolates, but with lower percentages. Most of resistant *E. coli*, including multidrug-resistant strains, were isolated from poultry fecal samples. More than 30% of samples were positive for *E. coli* resistant to third-generation cephalosporins or quinolone-based antibiotics. All isolated *Salmonella* were pan-susceptible. These results give an insight into the status of antibiotic resistance in food animals in Rwanda, as well as a call for further research. Also, the findings indicated a need for antibiotic stewardship and antimicrobial resistance surveillance initiatives.

**Abstract:**

In Rwanda, information on antibiotic resistance in food animals is scarce. This study was conducted to detect and phenotypically characterize antibiotic-resistant *Escherichia coli* and *Salmonella* in feces of cattle, goats, pigs, and poultry in the East province of Rwanda. We isolated non-type-specific (NTS) *E. coli* and *Salmonella* using plain culture media. In addition, we used MacConkey agar media supplemented with cefotaxime at 1.0 μg/mL and ciprofloxacin at 0.5 μg/mL to increase the probability of detecting *E. coli* with low susceptibility to third-generation cephalosporins and quinolones, respectively. Antibiotic susceptibility testing was performed using the disk diffusion test. Among 540 NTS *E. coli* isolates, resistance to tetracycline was the most frequently observed (35.6%), followed by resistance to ampicillin (19.6%) and streptomycin (16.5%). Percentages of NTS *E. coli* resistant to all three antibiotics and percentages of multidrug-resistant strains were higher in isolates from poultry. All isolated *Salmonella* were susceptible to all antibiotics. The sample-level prevalence for resistance to third-generation cephalosporins was estimated at 35.6% with all third-generation cephalosporin-resistant *E. coli*, expressing an extended-spectrum beta-lactamase phenotype. The sample-level prevalence for quinolone resistance was estimated at 48.3%. These results provided a baseline for future research and the development of integrated surveillance initiatives.

## 1. Introduction

The factors influencing the emergence, propagation, and spread of bacteria resistant to antibiotics are complex and not fully understood [1]. Despite this uncertainty, antimicrobial administration to animals is an important determinant in the animal population-level burden of antimicrobial resistance (AMR). This justifies why scientists are encouraged to generate information in an effort to understand AMR and guide decision-making related to its control [2,3].

High levels of antimicrobial resistance in food-producing animals have been reported in African countries, with resistance to tetracyclines and penicillin being the most frequently observed [4,5]. In East African countries, most research on AMR has thus far been conducted in Kenya, Ethiopia, Uganda, and Tanzania, while in Rwanda and Burundi very little data are available [6]. In Tanzania and Uganda, levels of resistance to tetracycline, sulphamethoxazole/trimethoprim, and ampicillin among indicator *Escherichia coli* from food animals were reported to be high compared to levels of resistance to cefotaxime [7,8]. Some studies have linked high rates of antibiotic resistance in animals with antibiotic usage on farms. For instance, in Uganda, it was demonstrated that the occurrence of ampicillin resistance was significantly correlated with the usage of penicillin in livestock [8]. Besides livestock, antimicrobial resistance was reported in different wildlife at levels relatively lower or close to levels reported in humans and livestock [9,10,11,12]. Worryingly, the contact between wildlife, humans, and livestock is increasing. Consequently, there is a higher probability of dissemination of antimicrobial-resistant pathogens among ecosystems [10]. While some studies have failed to prove the transmission of antibiotic-resistant bacteria between wildlife and humans [12], other studies have established the role of wildlife in the dissemination of antibiotic-resistant bacteria. In Kenya, urban wildlife, such as birds and mammals, have been identified as conduits for the transmission of antibiotic-resistant bacteria to the wider environment [11].

In Rwanda, the limited data available on AMR are restricted to studies conducted to estimate levels of AMR among bacterial pathogens causing diseases in humans. The reported percentages of pathogens resistant to cephalosporins, ciprofloxacin, or gentamycin among patients indicate an increasing trend of antimicrobial resistance in hospitals [13,14,15,16,17,18,19,20]. For example, it was shown that the susceptibility of *E. coli* to gentamycin, colistin, imipenem, piperacillin, and nalidixic acid was decreasing with time [21]. Although these studies were solely hospital-based, they are indicative of a serious issue in Rwanda that needs attention. The exact evidence of transmission of antibiotic-resistant bacteria between humans and animals has not been established in the country. However, indicators are showing a possibility for pathogen transmission between animals and humans, mainly due to poor hygiene. High levels of meat contaminated with *Salmonella* were reported in Kigali [22], and insufficient safety measures were noted along the milk chain in Rwanda [23].

Among animals, however, only a few studies on the prevalence of AMR among bacteria isolated from food animals have been conducted [24]. The present study was conducted to address gaps in the information on AMR in food animals in the country.

In Rwanda, livestock production plays an important role in agricultural households as a source of food and income. In 2017, it was estimated that 62.6% of 2.1 million agricultural households were engaged in livestock [25]. Cattle are the food-producing animals owned by most agricultural households. They are followed by goats, poultry, pigs, sheep, and rabbits [25]. In 2018, the national livestock population (heads of animals) was estimated at 1,293,768 cattle; 2,731,795 goats; 601,836 sheep; 1,330,461 pigs; 1,264,734 rabbits; and 5,442,152 poultry [26]. The distribution of food-producing animals varies according to provinces. The latest national agriculture survey showed that most cattle, goats, and poultry were located in the East province, while most sheep were located in the North province and most pigs were in the South province [27].

Cattle meat is the most produced meat in Rwanda, followed by pork, chicken, goat, and sheep meat. Crossbreeds are increasing in cattle production, while local breeds are the most common in goat, sheep, and pig production. Even if 75% of the national chicken population is occupied by local breeds reared in the village chicken system, most of the chicken meat and eggs produced in Rwanda come from specialized broilers and layers, respectively [28]. In general, the current food animal production is dominated by a family-run production system without any specialization. There is still a lower number of specialized animal farms with intensive production of milk, meat, or eggs [28].

Livestock production contributes 4% to the total gross domestic product, and is one of the fastest-growing subsectors in Rwanda. Between 2012 and 2016, its growth was estimated at 8.3% [28]. This high performance is attributed to governmental efforts to improve and modernize the animal production sector through various programs, such as programs of intensification, the Girinka program (one cow per family), and small stock development.

Despite governmental efforts to improve animal production to contribute to food security and income generation, animal diseases represent an important constraint to the sector. Various infectious diseases, including bacterial diseases, prevail in food animals in Rwanda [29,30,31,32,33,34,35,36]. Some of the prevalent bacterial diseases include anthrax, bovine tuberculosis, brucellosis (*Brucella abortus, Brucella melitensis*), swine erysipelas, contagious bovine pleuropneumonia, contagious caprine pleuropneumonia, fowl typhoid, heartwater, and ovine epididymitis (*Brucella. Ovis*) [29]. High levels of morbidity and mortality in livestock are considered as major drivers of antibiotic use in animals. In addition, poor diagnostics and uncontrolled access to antibiotics can lead to antibiotics misuse [37,38]. Unfortunately, data on types and quantities of antibiotics used in food animals are missing.

The paucity of information on the occurrence and the magnitude of AMR in food animals makes it difficult to objectively design contextual strategies to prevent and control AMR in food animals in Rwanda.

The end-goal of this study was to address gaps in the information on AMR in food animals in Rwanda. Specifically, the study’s objective was to provide initial estimates of isolate- and sample-level prevalence of indicator *E. coli* and pathogenic *Salmonella* resistant to antibiotics among cattle, goats, pigs, and poultry feces in the East province of Rwanda.

## 2. Materials and Methods

### 2.1. Fecal Sample Collection

**Figure 1:**
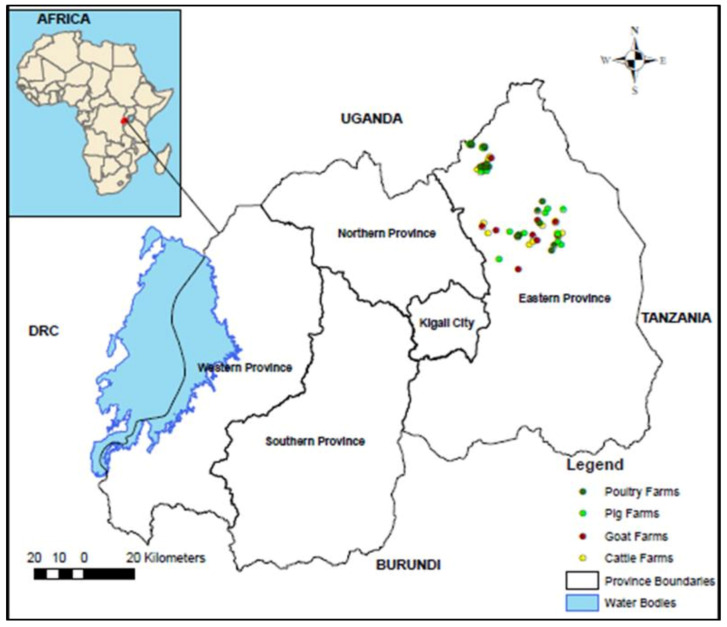
Location of visited farms in the East province of Rwanda. Approximate locations of visited farms were generated based on administrative units (cells) where farms were located.

From September to November 2019, a convenience sample size of 180 feces was collected from cattle, goats, pigs, and poultry in farms located in the East province of Rwanda (Figure 1). In general, visited farms were diverse in terms of animal population and production management. All cattle farms had between 15 and 30 animals, goat farms had between 15 and 50 animals, and pig farms had between 10 and 35 animals. All cattle, goat, and pig farms were non-specialized, intensive-production farms. They can be categorized as semi-intensive farms [38]. All poultry farms were large in terms of chicken population and were commercial farms with either broiler or layer production.

The northeast region of Rwanda has an established livestock farming culture and higher livestock density than other regions in Rwanda [27]. For each animal species, 15 farms, identified and recruited via snowball sampling [39], were visited. At each farm, three freshly voided feces were randomly collected in clean cups using clean spoons. In total, 45 fecal samples were collected per animal species. Samples were kept on ice in a cooler box and transported by car to the Rwanda Agriculture and Animal Resources Development Board’s microbiology laboratory in Kigali for processing.

The collection of fecal samples was done without any interaction with animals. Voided feces were collected from the floor. Therefore, ethical approval was not required.

### 2.2. Bacteria Isolation

The protocol used in this study was adapted from a protocol developed and field-tested on dairy faecal samples in Texas [40]. Briefly, 10 gr of each faecal sample was enriched in 90 mL of buffered peptone water (Hardy Diagnostics, Santa Maria, CA, USA) overnight. The obtained non-selective enrichment was used for bacterial isolation. Ten µL of the non-selective enrichment was streaked on plain (without antibiotics) MacConkey agar (Hardy Diagnostics, Santa Maria, CA, USA) for the isolation of non-type-specific (NTS) *E. coli*. Moreover, to increase the probability of isolating third-generation cephalosporin-resistant (3GCr) *E. coli* and quinolone-resistant (Qr) *E. coli*, a screening step was performed, using MacConkey agar containing cefotaxime (Acros Organics, Fair Lawn, NJ, USA) at 1.0 μg/mL and MacConkey agar containing ciprofloxacin (Acros Organics, Fair Lawn, NJ, USA) at 0.5 μg/mL, respectively [40,41]. In addition, 1 mL of the non-selective enrichment was transferred into 9 mL of Rappaport–Vassiliadis Salmonella (Becton Dickinson, Franklin Lakes, NJ, USA) and 9 mL of Tetrathionate (Becton Dickinson, Franklin Lakes, NJ, USA) broths for *Salmonella*-selective enrichments. Thereafter, 10 µL of each of the selective enrichments was streaked on plain brilliant green sulfa agar (Becton Dickinson, Franklin Lakes, NJ, USA) and plain xylose lysine tergitol4 agar (Becton Dickinson, Franklin Lakes, NJ, USA) for the isolation of *Salmonella*. Based on morphological appearance, three typical *E. coli* colonies grown on plain MacConkey agar and one colony from each of the MacConkey agar containing antibiotics were selected for confirmation as *E. coli* using the indole spot test (Hardy Diagnostics, Santa Maria, CA, USA). For each sample, two colonies with morphology typical to *Salmonella* were selected and confirmed as *Salmonella* by detection of the *invA* gene using loop-mediated isothermal amplification (LAMP)m as described by Hara-kudo [42]. The LAMP was performed using the WarmStart Colorimetric LAMP 2X Master Mix (New England Biolabs, Ipswich, MA, USA) following the manufacturer guidelines. The primers used were developed by Hara-kudo [42], and they are listed in Table S1 (Supplementary Materials).

### 2.3. Phenotypic Antibiotic Susceptibility Testing

Phenotypic antibiotic susceptibility testing was performed using the disk diffusion method, according (where applicable) to standards [43]. All bacterial isolates confirmed as *E. coli* and *Salmonella* were tested against 12 antibiotic drugs (Becton Dickinson, Franklin Lakes, NJ, USA) belonging to seven antibiotic classes: amoxicillin–clavulanic acid (AMC, 20/10 μg), ampicillin (AMP, 10 μg), azithromycin (AZI, 15 μg), cefoxitin (FOX, 30 μg), ceftriaxone (CRO, 30 μg), chloramphenicol (CHL, 30 μg), ciprofloxacin (CIP, 5 μg), colistin (COL, 10 μg), meropenem (MER, 10 µg), nalidixic acid (NAL, 30 µg), streptomycin (STR, 10 µg), and tetracycline (TET, 30 μg). The recorded inhibition zone diameters were interpreted using breakpoints from the Clinical and Laboratory Standards Institute (CLSI) guideline. A bacterial isolate was considered as likely to be resistant to colistin if the inhibition zone diameter was less than or equal to 11 mm [44]. Resistance to three or more antibiotic classes was the criteria used to classify bacterial isolates as multidrug-resistant (MDR) [45].

Furthermore, the combination disk test [43] was used to detect bacterial isolates, producing extended-spectrum beta-lactamases (ESBLs) among 3GCr isolates. A second panel of antibiotics (Becton Dickinson, Franklin Lakes, NJ, USA) was used: amikacin (AMK, 30 μg), cefazolin (CFZ, 30 μg), cefepime (FEP, 30 μg), cefotaxime (CTX, 30 μg), cefotaxime–clavulanic acid (CTX-CLA, 30/10 µg), ceftazidime (CAZ, 30 μg), ceftazidime–clavulanic acid (CAZ-CLA, 30/10 µg), fosfomycin (FOS, 200 μg), gentamicin (GEN, 10 μg), imipenem (IMP, 10 μg), sulfisoxazole (SSS, 300μg), and trimethoprim/sulfamethoxazole (SXT, 1.25/23.75 μg).

*E. coli* ATCC 25,922 was used for quality control purposes.

### 2.4. Statistical Analysis

Statistical analyses were performed using R software version 3.0.0 (R Core Team, Vienna, Austria). The Fisher’s exact test of independence was used for comparison of the prevalence of resistant bacteria among food animal species, with a *p*-value threshold of 0.05. The Wilson score method was used to calculate 95% confidence intervals (95% CI).

## 3. Results

Five hundred and forty NTS *E. coli* were isolated from plain MacConkey agar, with 135 isolates arising from each animal species. The screening process resulted in 69 samples with presumptive 3GCr *E. coli* and 160 samples with presumptive Qr *E. coli*. Four *Salmonella* isolates were recovered from only two pig fecal samples (*n* = 2 of 180 samples; 1.1%).

### 3.1. Antibiotic Susceptibility

At the isolate level, among the 540 NTS *E. coli*, the prevalence of isolates resistant to tetracycline was the most observed (Table 1).

The isolate-level prevalence of NTS *E. coli* resistant to ampicillin, quinolones, streptomycin, and tetracycline differed statistically (*p* < 0.05) among animal species, and were higher among isolates from poultry samples than from all other food animal species (Table 2).

**Figure 2:**
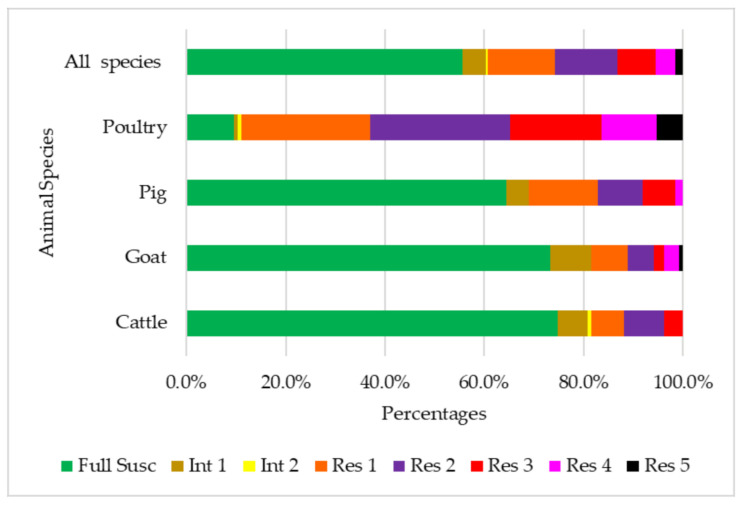
Multidrug-resistant *E. coli* isolated from the feces of food animals in the East province of Rwanda. The total number of bacterial isolates per animal species was 135. Full Susc: fully susceptible; Int 1: fully susceptible, but intermediate to one antibiotic class; Int 2: fully susceptible, but intermediate to two antibiotic classes; Res 1: resistant to one antibiotic class; Res 2: resistant to two antibiotic classes; Res 3: resistant to three antibiotic classes; Res 4: resistant to four antibiotic classes; Res 5: resistant to five antibiotic classes.

In total, 71 of the 540 NTS *E. coli* (13.1%) were of a multidrug resistance phenotype. The prevalence of MDR NTS *E. coli* was statistically different among food animal species (*p* < 0.05), with isolates from poultry samples (34.8%) being the highest vis-à-vis the prevalence of MDR NTS *E. coli* among isolates from the other three animal species (i.e., 3.7% in cattle, 5.9% in goats, and 8.1% in pigs) (Figure 2).

The four *Salmonella* isolates were all pan-susceptible to the 12 antibiotics tested.

**Figure 3:**
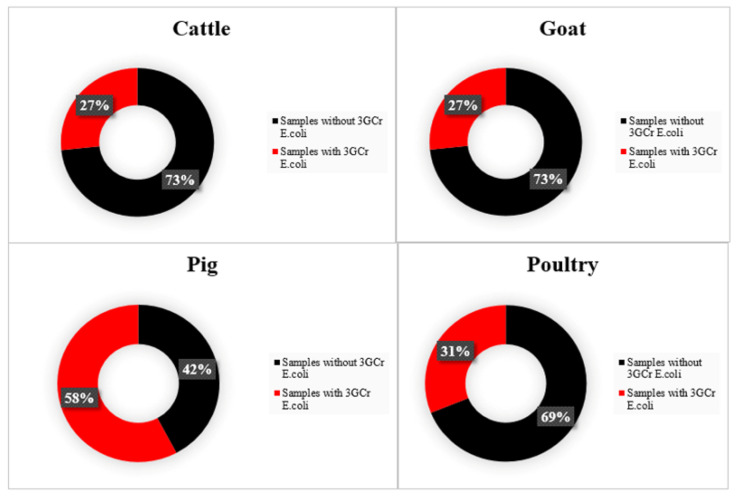
Recovery rates of 3GCr *E. coli* from cattle, goats, pigs, and poultry fecal samples in the East province of Rwanda. The total number of samples for each animal species was 45. 3GCr: third-generation cephalosporin-resistant.

Among 69 *E. coli* isolates screened on MacConkey agar containing cefotaxime, 64 were confirmed to be resistant to ceftriaxone. The sample-level prevalence for third-generation cephalosporin resistance among all food animal species was estimated at 35.6% (95% CI: 28.9–42.8%). The recovery of confirmed 3GCr *E. coli* was significantly different among food animal species (*p* < 0.05), with pigs having the highest (57.8%) in comparison to samples from cattle, goats, and poultry (Figure 3).

Confirmed 3GCr *E. coli* (*n* = 64) exhibited resistance mostly to ampicillin, followed by tetracycline (Table 3).

**Figure 4:**
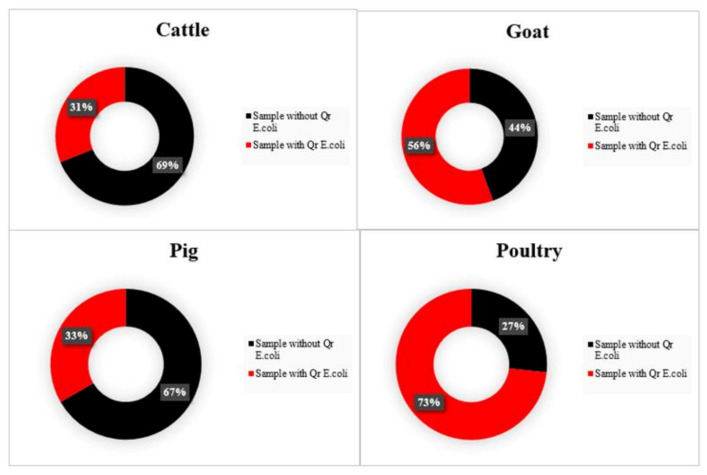
Recovery rates of Qr *E. coli* from cattle, goats, pigs, and poultry fecal samples in the East province of Rwanda. The total number of samples for each animal species was 45. Qr: quinolone resistant.

From 160 *E. coli* isolates screened on MacConkey agar with ciprofloxacin, 87 were confirmed as resistant to quinolone-based antibiotics (nalidixic acid or ciprofloxacin). The sample-level prevalence for quinolone resistance was estimated at 48.3% (95% CI: 41.1–55.6%). The recovery of confirmed Qr *E. coli* differed significantly among the food animal species (*p* < 0.05), with samples from poultry (73.3%) and goats (55.6%) higher in comparison to samples from cattle and pigs (Figure 4).

The proportion of Qr *E. coli* resistant to tetracycline was high (Table 4).

### 3.2. E. coli Producing ESBLs and Patterns of Resistance to Quinolones

In total, 72 isolates were confirmed as 3GCr *E. coli*. Among them, 64 were isolated from MacConkey agar with cefotaxime, while eight were isolated in MacConkey agar with ciprofloxacin. Most 3GCr *E. coli* had a phenotypic resistance to cefazolin and sulfisoxazole on the second panel of disks. It was found that all of the isolated 3GCr *E. coli* were phenotypically ESBL producers, except for one isolate that remains unclassified (Table 5).

In total, 141 *E. coli* isolates were resistant to quinolone-based antibiotics. Among them, 87 were isolated from MacConkey agar with ciprofloxacin, 19 were isolated from MacConkey agar with cefotaxime, and 35 were isolated from plain MacConkey agar. Among the 141 Qr *E. coli*, 24.8% were susceptible to ciprofloxacin but resistant to nalidixic acid, 19.9% were intermediate to ciprofloxacin and resistant to nalidixic acid, while 55.3% were resistant to ciprofloxacin and nalidixic acid concurrently.

## 4. Discussion

This study revealed that resistance to tetracycline was the most prevalent among NTS *E. coli* isolated from food animals, followed by resistance to ampicillin and streptomycin. This is similar to what was reported in some countries of East Africa, such as Uganda, Tanzania, and Kenya, where resistance to tetracycline, ampicillin, and streptomycin were ranked among the most predominant in food animals [7,8,46]. Resistance to oxytetracycline was the most common resistance among *E. coli* from chicken in Thailand, Indonesia, and Vietnam [47]. Our results are not that different from the global trend of antimicrobial resistance in animals in low- and middle-income countries. Van Boeckel and collaborators reported that resistance to tetracycline, sulfonamide, and penicillin were the most frequently observed in animals in these countries [48]. We also noted that most of the resistant NTS *E. coli*, specifically those resistant to tetracycline and multidrug-resistant strains, were isolated from poultry fecal samples. Even if the most common antibiotic used in food animals in Rwanda is not documented, situations reported in adjacent countries may be comparable for farmers in Rwanda. It is reported that tetracycline is the most widely used antibiotic in food animals in Tanzania and Kenya [49,50]. This is also true for other African countries, where it has been reported that tetracycline, aminoglycoside, and penicillin groups are the most commonly used antibiotics in animals [5]. In the majority of low- and middle-income countries, tetracycline, sulfonamide, and penicillin antibiotic groups have been reported as the most commonly used [48]. In Rwanda, oxytetracycline and peni-streptomycin were identified as the main antibiotics recognized or known by farmers [38]. In the same study, poultry farmers were predicted to have a moderate level of antibiotic use in their animals, where they could use antibiotics on regular basis for disease prevention or growth promotion [38]. If tetracycline is heavily used in food animals, specifically in poultry, in the study area, this would help to explain the high level of resistance to tetracycline observed among NTS *E. coli* isolated from poultry. We recommend further studies to establish the types and amounts of antibiotics used in food-producing animals to address this hypothesis.

In our study, the recovery of *Salmonella* was low. The detection and isolation of *Salmonella* can be challenging. It is recommended to combine various *Salmonella* selective media for efficient detection [51]. In this study, two *Salmonella*-selective broths (i.e., Rappaport–Vassiliadis Salmonella and Tetrathionate) were used to selectively grow *Salmonella* in samples before isolation on two *Salmonella*-selective agar-based media. A study has demonstrated that Rappaport–Vassiliadis and Tetrathionate are effective media to isolate *Salmonella* [52]. On the other hand, another study demonstrated a relatively low ability of Rappaport–Vassiliadis medium to recover *Salmonella* when used alone [53]. The low recovery of *Salmonella* in our study could also indicate a low prevalence of *Salmonella* in food animals. In some countries, it is documented that *Salmonella* prevalence in animals varies with seasons [54,55]. Unfortunately, due to the lack of studies involving the isolation of *Salmonella* in food animals in Rwanda, it remains difficult to explain the low recovery rate observed in this study. With such a low recovery rate of only four strains of pan-susceptible *Salmonella*, it remains impossible to provide an overview of antibiotic resistance among *Salmonella* in food animals in Rwanda.

It was found that 35.6% of samples were positive for 3GCr *E. coli*, even though in many East African countries and the majority of low- and middle-income countries, third-generation cephalosporin antibiotics are not widely used in food animals [49,50]. Direct selection pressure seems unlikely; therefore, exploration as to the likely co-selection pressures [56,57] is needed. Further characterization of 3GC resistance genes would be needed to confirm this, along with other resistance genes that might be co-located on mobilizable genetic elements. In low- and middle-income countries, the rate of bacteria resistant to third- and fourth-generation cephalosporins in animals was reported to be moderate, ranging between 10% and 40%. Comparable to results reported in Thailand [58], the recovery rate of 3GCr *E. coli* was higher in pig fecal samples. Similar to results reported in Tanzania [7], all 3GCr *E. coli* isolated in this study presented a phenotype of ESBL production. Additionally, it was noted that most of 3GCr *E. coli* were resistant to cefotaxime rather than ceftazidime. This may suggest that *bla*_CTX-M_ genes are the most prevalent in *E. coli* from food animals in the study area. Previous studies [59] have reported that *bla*_CTX-M_ genes generally confer lower resistance to ceftazidime than to cefotaxime, which is borne out in our study (Table 5).

Furthermore, our recovery of Qr *E. coli* (48.3%) was within the estimated range for quinolone resistance in animals in low- and middle-income countries (20% to 60%) [48]. The phenotype of quinolone resistance in *E. coli* can help in the prediction of the resistance mechanism involved [60,61]. Based on previously reported observations [60,61], we predicted that most of Qr *E. coli* isolated in this study had mutated *gyrA* and *parC* genes. This prediction was based on the fact that the majority of Qr *E. coli* isolated was resistant to all quinolone-based antibiotics tested (nalidixic acid and ciprofloxacin). In addition, we predicted that Qr *E. coli* classified as susceptible or intermediate to ciprofloxacin, but with a resistance to nalidixic acid, had a mutation in the *gyrA* gene or *parC* gene, or else had the *qnr* gene. In Vietnam, a study demonstrated that 30 of 33 *E. coli* resistant to nalidixic acid but intermediate to ciprofloxacin had a *qnr* gene [62].

Several *E. coli* isolates from the feces of food animals in the present study were resistant to various antibiotics, including critically important antibiotics for human and veterinary medicine [63,64]. Antibiotic-resistant bacteria or genes of resistance in food animals can be transmitted to humans directly through contact with animals, or indirectly through the consumption of animal products or a contaminated environment [65]. The results of this study indicate a need to use a one health approach to control the challenge of antimicrobial resistance in Rwanda.

The absence of data on AMR in food animals in Rwanda needs to be addressed urgently. Consequently, the present results are made public to provide preliminary information on antibiotic resistance in food animals in Rwanda. Nevertheless, the next step of this study will be the exploration of genes and genetic determinants of antibiotic resistance among the isolated bacteria.

## 5. Conclusions

Overall, this study provides an overview of the distribution of AMR among food animals in the East province of Rwanda. This early insight feeds a call for more research that can cover the whole country and entire food chains. These findings can also serve as a basis of design upon which an integrated AMR surveillance system in Rwanda can be developed.

## Figures and Tables

**Table 1:** Susceptibility to antibiotics of *E. coli* (*n* = 540) from food animals in the East province of Rwanda.

ATB	R		I	S	Distribution (Number) in Each Inhibition Zone: Diameter (in mm)																				
	%	95% CI	%	%	0	7	8	9	10	11	12	13	14	15	16	17	18	19	20	21	22	23	24	25	>25
AMC	0.7	0.3–1.9	1.1	98.1				1	1	1	1		1	2	1	2	9	12	33	63	87	90	80	69	87
AMP	19.6	16.5–23.2	2.0	78.3	99	1	1	2	1	2			1	5	5	9	38	57	81	73	64	53	24	12	12
AZI	1.1	0.5–2.4		98.9				1	3		2	2	5	7	6	11	35	40	39	44	64	70	66	1	144
FOX	0.7	0.3–1.9	0.6	98.7	3				1						1	2	1	2	3	17	41	72	101	105	191
CRO	0.0	0.0–0.7	0.4	99.6																1	1		3	8	527
CHL	3.3	2.1–5.2	0.2	96.5	12	2	2		2							1	1	1	1	1	9	23	40	65	380
CIP	3.1	2.0–5.0	1.7	95.2	3				1	2	3	3	4	1	2	2	2	1	2	2	3	4	6	13	486
COL	2.0	1.1–3.6	40.4	57.6					1	10	80	138	156	106	39	9	1								
MER	0.0	0.0–0.7	0.2	99.8																1		1	1	7	530
NAL	6.5	4.7–8.9	4.3	89.3	25		1	2	4		2	1	3		7	7	6	8	8	23	24	53	66	72	228
STR	16.5	13.6–19.8	6.1	77.4	57	6	4	11	4	7	5	7	21	55	101	88	89	52	21	5	4	2	1		
TET	35.6	31.6–39.7	1.3	63.1	101	22	32	23	11	3	5	1	1				1	4	13	35	44	47	58	50	89

*E. coli* isolated from plain MacConkey agar. ATB: antibiotic, R: resistance, I: intermediate, S: susceptible, 95% CI: 95% confidence intervals, AMC: amoxicillin-clavulanic acid, AMP: ampicillin, AZI: azithromycin, FOX: cefoxitin, CRO: ceftriaxone, CHL: chloramphenicol, CIP: ciprofloxacin, COL: colistin, MER: meropenem, NAL: nalidixic acid, STR: streptomycin, TET: tetracycline. Interpreted according to clinical breakpoints for Enterobacteriaceae [43]: dark grey fields present frequencies of resistant isolates, fields in light grey with borders present frequencies of intermediate isolates, and white fields present frequencies of susceptible isolates. COL: clinical breakpoints [44].

**Table 2:** Resistance to antibiotics among *E. coli* from food animals in the East province of Rwanda.

	Cattle (*n* = 135)	Goats (*n* = 135)	Pigs (*n* = 135)	Poultry (*n* = 135)	Total (*n* = 540)
Antibiotic	Number (%)	Number (%)	Number (%)	Number (%)	Number (%)
Amoxicillin–clavulanic acid	1 (0.7)	2 (1.5)	0 (0.0)	1 (0.7)	4 (0.7)
Ampicillin *	6 (4.4)	10 (7.4)	17 (12.6)	73 (54.1)	106 (19.6)
Azithromycin	0 (0.0)	1 (0.7)	1 (0.7)	4 (3.0)	6 (1.1)
Cefoxitin	1 (0.7)	2 (1.5)	1 (0.7)	0 (0.0)	4 (0.7)
Ceftriaxone	0 (0.0)	2 (1.5)	0 (0.0)	0 (0.0)	2 (0.4)
Chloramphenicol *	0 (0.0)	3 (2.2)	3 (2.2)	12 (8.9)	18 (3.3)
Ciprofloxacin *	1 (0.7)	2 (1.5)	0 (0.0)	14 (10.4)	17 (3.1)
Colistin	2 (0.4)	5 (0.9)	2 (0.4)	2 (0.4)	11 (2.0)
Meropenem	0 (0.0)	0 (0.0)	0 (0.0)	0 (0.0)	0 (0.0)
Nalidixic acid *	1 (0.7)	3 (2.2)	0 (0.0)	31 (23.0)	35 (6.5)
Streptomycin *	15 (11.1)	12 (8.9)	18 (13.3)	44 (32.6)	89 (16.5)
Tetracycline *	22 (16.3)	19 (14.1)	36 (26.7)	115 (85.2)	192 (35.6)
Pan-susceptible *	101 (74.8)	99 (73.3)	87 (64.4)	13 (13.6)	300 (55.6)

Within any row, an asterisk indicates a statistically significant difference in the prevalence of resistant NTS *E. coli* among animal species (*p* < 0.05). *n* = total number of NTS *E. coli*. Pan-susceptible are isolates susceptible to all antibiotics (excludes isolates classified as either intermediate or resistant). *E. coli* presented in this table were isolated in plain MacConkey agar.

**Table 3:** Susceptibility to antibiotics of *E. coli* resistant to third-generation cephalosporins (*n* = 64) from food animals in the East province of Rwanda.

ATB	R		I	S	Distribution (Number) in Each Inhibition Zones: Diameters (in mm)																				
	%	95% CI	%	%	0	7	8	9	10	11	12	13	14	15	16	17	18	19	20	21	22	23	24	25	>25
AMC	1.6	0.3–8.3	7.8	90.6				1					1	1	1	2	8	10	10	11	11	5	2	1	
AMP	100.0	94.3–100.0	0	0	64																				
AZI	14.1	7.6–24.6		85.9	3			1	1		4			1	1		4	1	6	2	8	11	5	4	12
FOX	1.6	0.3–8.3	0	98.4	1													1	2	3	7	11	14	12	13
CRO	100.0	94.3–100.0	0	0	18	1	2	12	8	16	5	2													
CHL	0.0	0.0–5.7	0	100																1	2		1	6	54
CIP	15.6	8.7–26.4	4.7	79.7	8			1			1				1	2				2	3	2	4	9	31
COL	0.0	0.0–5.7	32.8	67.2							8	13	22	17	4										
MER	0.0	0.0–5.7	0	100																			1	2	61
NAL	29.7	19.9–41.8	6.3	64.1	15		1	2				1	1	2			1	8	2	9	5	10	3		4
STR	84.4	73.6–91.3	4.7	10.9	26	9	9	6	3	1	1		2	3	2	2									
TET	87.5	77.2–93.5	0	12.5	22	4	14	11	2	3										1	1	2	2	2	

*E. coli* isolated on MacConkey agar containing cefotaxime at 1.0 µg/mL. ATB: antibiotic, R: resistance, I: intermediate, S: susceptible, 95% CI: 95% confidence intervals, AMC: amoxicillin–clavulanic acid, AMP: ampicillin, AZI: azithromycin, FOX: cefoxitin, CRO: ceftriaxone, CHL: chloramphenicol, CIP: ciprofloxacin, COL: colistin, MER: meropenem, NAL: nalidixic acid, STR: streptomycin, TET: tetracycline. Interpreted according to clinical breakpoints for Enterobacteriaceae [43]: dark grey fields present frequencies of resistant isolates, fields in light grey with borders present frequencies of intermediate isolates, and white fields present frequencies of susceptible isolates. COL: clinical breakpoints [44].

**Table 4:** Susceptibility to antibiotics of *E. coli* resistant to quinolones (*n* = 87) from food animals in the East province of Rwanda.

ATB	R		I	S	Distribution (Number) in Each Inhibition Zones: Diameters (in mm)																				
	%	95% CI	%	%	0	7	8	9	10	11	12	13	14	15	16	17	18	19	20	21	22	23	24	25	>25
AMC	0	0.0–4.2	2.3	97.7												2	8	6	16	16	13	11	8	5	2
AMP	55.2	44.7–65.2	1.1	42.5	46	1					1				1	5	3	4	9	11	3	1	1		1
AZI	13.8	8.1–22.6		86.2					2	5	5	1	5	6	1	3	8	5	12	7	7	7	5	4	4
FOX	1.1	0.2–6.2	2.3	96.6							1					2		5	4	10	15	10	15	14	11
CRO	1.1	0.2–6.2	0	98.9						1												2	1	3	80
CHL	17.2	10.7–26.5	0	82.8	10		2	2			1							1	3	4		5	11	10	38
CIP	58.6	48.1–68.4	20.7	19.5	24			2		3	8	8	4	2	1	4	5		8	2	3	5	4	3	1
COL	3.4	1.2–9.7	52.9	43.7						3	16	30	30	5	2	1									
MER	0	0.0–4.2	0	100.0																			1	4	82
NAL	100.0	95.8–100.0	0	0	63		3	2	8	4	4	3													
STR	48.3	38.1–58.6	13.8	37.9	32	4	3	1	1	1	1	2	9	9	12	4	4	3	1						
TET	95.4	88.8–98.2	0	4.6	53	9	7	7	5	2												2	2		

*E. coli* isolated on MacConkey agar containing ciprofloxacin at 0.5 µg/mL. ATB: antibiotic, R: resistance, I: intermediate, S: susceptible, 95% CI: 95% confidence intervals. AMC: amoxicillin–clavulanic acid, AMP: ampicillin, AZI: azithromycin, FOX: cefoxitin, CRO: ceftriaxone, CHL: chloramphenicol, CIP: ciprofloxacin, COL: colistin, MER: meropenem, NAL: nalidixic acid, STR: streptomycin, TET: tetracycline. Interpreted according to clinical breakpoints for Enterobacteriaceae [43]: dark grey fields present frequencies of resistant isolates, fields in light grey with borders present frequencies of intermediate isolates, and white fields present frequencies of susceptible isolates. COL: clinical breakpoints [44].

**Table 5:** Susceptibility to the second panel of antibiotics of all *E. coli* resistant to third-generation cephalosporins (*n* = 72) from food animals in the East province of Rwanda.

ATB	R		I	S	Distribution (Number) in Each Inhibition Zones: Diameters (in mm)																							
	%	95% CI	%	%	0	7	8	9	10	11	12	13	14	15	16	17	18	19	20	21	22	23	24	25	26	27	28	>28
AMK	0	0.0–5.1	0	100.0													1	3	5	10	6	17	14	8	5	1		2
CFZ	98.6	92.5–99.8		1.4	71															1								
FEP	45.8	34.8–57.3	50	4.2	2			2	2		2	2	5	4	2	7	5	11	12	9	1	1	2	1				2
CTX	98.6	92.5–99.8	0	1.4	34	2	6	6	8	7	3	2	1		1	1												1
CTX-CLA													1				2	3	1	3	1	5	5	5	10	8	13	15
CAZ	31.9	22.3–43.4	43.1	25	1									3	7	12	14	11	6	5	5	4	1	2			1	
CAZ-CLA												1									1		1	3	12	13	14	27
FOS	0	0.0–5.1	0	100.0																	2	5	6	9	5	10	9	26
GEN	6.9	3.0–15.2	0	93.1			1	3		1						1	1	7	16	10	16	10	4	1	1			
IMP	0	0.0–5.1	0	100.0																		2	3	5	7	9	7	39
SSS	95.8	88.5–98.6	0	4.2	67	1	1														2							1
SXT	88.9	79.6–94.3	0	11.1	63	1										1	1				1	1	1			1		2
ESBL	98.6	92.5–99.8																										
AmpC	0	0.0–5.1																										

ATB: antibiotic, R: resistance, I: intermediate, S: susceptible, 95% CI: 95% confidence intervals, AMK: amikacin, CFZ: cefazolin, FEP: cefepime, CTX: cefotaxime, CAZ: ceftazidime, FOS: fosfomycin, GEN: gentamicin, IMP: imipenem, SSS: sulfisoxazole, SXT: trimethoprim/sulfamethoxazole. Interpreted according to clinical breakpoints for Enterobacteriaceae [43]: dark grey fields present frequencies of resistant isolates, fields in light grey with borders present frequencies of intermediate isolates, and white fields present frequencies of susceptible isolates. CTX-CLA and CAZ-CLA do not have Clinical and Laboratory Standards Institute (CLSI) clinical breakpoints. ESBL represents the percentage of *E. coli* with an extended-spectrum beta-lactamase (ESBL) phenotype. AmpC represents the percentage *E. coli* with an AmpC beta-lactamases phenotype.

## Data Availability

The data presented in this study are available on request from the corresponding author.

## Supplementary Materials

The following are available online at https://www.mdpi.com/article/10.3390/ani11041013/s1, Table S1: List of primers used in the Loop-mediated isothermal amplification to detect *invA* gene of *Salmonella*.
