# Supplementary material for: Antibiotic-Resistant Escherichia coli and Salmonella from the Feces of Food Animals in the East Province of Rwanda
Source: Animals (Basel). 2021 Apr 3;11(4):1013. doi: 10.3390/ani11041013 (PMC8067188; doi:10.3390/ani11041013)
Supplement: Supplementary file 1 [file animals-11-01013-s001.pdf]

Table S1: List of primers used in the Loop-mediated isothermal amplification to detect *invA* gene of *Salmonella*

PRIMER	Sequence (5′–3′)
FIP	GACGACTGGTACTGATCGATAGTTTTTCAACGTTTCCTGCGG
BIP	CCGGTGAAATTATCGCCACACAAAACCCACCGCCAGG
F3	GGCGATATTGGTGTTTATGGGG
B3	AACGATAAACTGGACCACGG
LOOP F	GACGAAAGAGCGTGGTAATTAAC
LOOP B	GGGCAATTCGTTATTGGCGATAG

Primers developed by Hara-kudo (2005).

Hara-kudo, Y. Loop-Mediated Isothermal Amplification for the Rapid Detection of Salmonella. *FEMS Microbiol. Lett.*, **2005**, *253*, 155–161. https://doi.org/10.1016/j.femsle.2005.09.032.
